# Supplementary material for: Influence of 4-week lower extremity high-intensity interval training on energy metabolism and maximal oxygen uptake of elite swimmers
Source: PeerJ. 2025 Jul 31;13:e19788. doi: 10.7717/peerj.19788 (PMC12318499; doi:10.7717/peerj.19788)
Supplement: Supplemental Information 3 [file peerj-13-19788-s003.docx]

**The Influence of 4-week Lower Extremity High-Intensity Interval Training on Energy Metabolism and Maximal Oxygen Uptake of Elite Swimmers**

Hello, we are a research team from Shanghai University of Sport, currently conducting a study on the effects of a 4-week lower-limb high-intensity interval training (HIIT) program on the energy metabolism and maximal oxygen uptake (VO₂max) of elite swimmers. Our research investigates how lower-limb HIIT influences different energy metabolism pathways, including phosphagen, glycolytic, and aerobic systems, and assesses its potential benefits for optimizing competitive swimming performance. Through this experiment, we aim to provide empirical evidence for improving aerobic and anaerobic endurance in swimmers and optimizing HIIT-based training strategies.

Principal Investigator: Chao Chen、Rangxi Jin、Junhui Sun, Na Jiang

**Participation Requirements:**

Inclusion Criteria:

1. Registered competitive swimmers from Shanghai University of Sport.

2. Aged 18–25 years with at least 5 years of systematic swimming training.

3. No major injuries or illnesses in the past 6 months that could impact training.

4. Participants must be in good physical and mental condition.

Exclusion Criteria:

- History of cardiovascular, respiratory, or musculoskeletal disorders.
- Any recent injury that may interfere with high-intensity training.
- Individuals currently undergoing rehabilitation or restricted from lower-limb intensive training.

**Experimental Content:**

- Participants will be randomly assigned to either the HIIT experimental group or the control group.
- The HIIT group will undergo a structured 4-week lower-limb high-intensity interval training program (3 sessions per week, 60 minutes per session).
- The control group will maintain their regular training regimen.
- Testing will include Lode lower-limb power cycling tests, gas metabolism analysis (VO₂max), and blood lactate measurement.

**Experiment Time:**

- **Duration:** 4 weeks (12 training sessions)
- **Each session:** 60 minutes (including warm-up, main training, and cool-down)
- **Testing time:** Approximately 90 minutes per session (pre-test and post-test assessments)

**Testing Time and Location:**

- Location: Shanghai University of Sport – Physical Training Research Center. Testing Period: April 2024 – June 2024.

**Experimental Precautions:**

- The training and testing procedures have been approved by the Ethics Committee of Shanghai University of Sport (102772021RT031).
- All participants will receive detailed instructions and be monitored throughout the study.
- This study involves standard lower-limb HIIT training on cycling ergometers, and all procedures are non-invasive.
- Possible risks: Participants may experience temporary muscle fatigue or mild soreness, which is a normal physiological response to high-intensity training.

**Registration Method:**

Students interested in participating in this study can send a text message to 15000855626 (Chao Chen) / 15868655635 (Rangxi Jin) or add the WeChat account (15000855626 / 15868655635). To register, please include the message "Participate in the experiment," and a staff member will contact you.
